# P163: Patients for patient safety: translating anecdote to evidence base

**DOI:** 10.1186/2047-2994-2-S1-P163

**Published:** 2013-06-20

**Authors:** M Murphy

**Affiliations:** 1 Patients for Patient Safety, WHO, Geneva, Switzerland

## Introduction

Capturing the “patient experience” in a manner that supports healthcare in providing quality care in a consistent manner while also effecting necessary change, continues to be a challenge for patients and patient organisations. The patient experience is often described as “the patient story” – an expression which, in itself, can sometimes detract from the perceived value of what is being offered as an accurate representation of the reality in relation to how care is actually delivered. Particularly in the area of education, the articulation of these experiences by “real people”, patients and family members, elevates the “story” to being an effective learning tool. This supports the didactic course material and promotes sustainable culture change to provide care that is truly patient-centred, especially in the areas of communication and adherence to guidance and protocols.

## Methods

Feedback from students, those engaged in continuing professional development, frontline staff, and policy makers shows that exposure to the patient experience directly from the patient is not alone welcomed, but is valued as an adjunct to the formal processes and is considered to be motivational in relation to enhancing the quality of clinical practice.

## Results

Healthcare is informed and monitored by clinical audit, process audit, and outcome audit. There is a singular absence of patient experience audit. Examples of two incidents of patient experiences show that the absence of the patient experience audit deprived healthcare of achieving a true reflection of whether adherence to guidance and protocols exists outside the audit process in the everyday delivery of care.

## Conclusion

Part of the process involves structuring the patient experience to optimum benefit, e.g., providing a chronology of events, identifying shortcomings, offering solutions. The resultant change in dynamics means that the patient and his/her unique experience are accepted as further and hitherto untapped resources to be harnessed in all areas of activity, such as policy making, education, research, and regulation.

## Disclosure of interest

None declared

